# Intraclonal genome diversity of *Pseudomonas aeruginosa* clones CHA and TB

**DOI:** 10.1186/1471-2164-14-416

**Published:** 2013-06-22

**Authors:** Oliver KI Bezuidt, Jens Klockgether, Sylvie Elsen, Ina Attree, Colin F Davenport, Burkhard Tümmler

**Affiliations:** 1 Klinische Forschergruppe, Klinik für Pädiatrische Pneumologie, Allergologie und Neonatologie, Medizinische Hochschule Hannover, Hannover D-30625, Germany; 2 Bioinformatics and Computational Biology Unit; Department of Biochemistry, University of Pretoria, Pretoria, South Africa; 3 INSERM, UMR-S 1036, Biology of Cancer and Infection, Grenoble, France; 4 CNRS, ERL 5261, Bacterial Pathogenesis and Cellular Responses, Grenoble, France; 5 UJF-Grenoble 1, Grenoble F-38041, France; 6 6CEA, DSV/iRTSV, Grenoble F-38054, France; 7 Biomedical Research in Endstage and Obstructive Lung Disease Hannover (BREATH), Member of the German Center for Lung Research, Hannover, Germany

**Keywords:** *Pseudomonas aeruginosa*, Microevolution, Habitat adaptation, Genome diversity

## Abstract

**Background:**

Adaptation of *Pseudomonas aeruginosa* to different living conditions is accompanied by microevolution resulting in genomic diversity between strains of the same clonal lineage. In order to detect the impact of colonized habitats on *P. aeruginosa* microevolution we determined the genomic diversity between the highly virulent cystic fibrosis (CF) isolate CHA and two temporally and geographically unrelated clonal variants. The outcome was compared with the intraclonal genome diversity between three more closely related isolates of another clonal complex.

**Results:**

The three clone CHA isolates differed in their core genome in several dozen strain specific nucleotide exchanges and small deletions from each other. Loss of function mutations and non-conservative amino acid replacements affected several habitat- and lifestyle-associated traits, for example, the key regulator GacS of the switch between acute and chronic disease phenotypes was disrupted in strain CHA. Intraclonal genome diversity manifested in an individual composition of the respective accessory genome whereby the highest number of accessory DNA elements was observed for isolate PT22 from a polluted aquatic habitat. Little intraclonal diversity was observed between three spatiotemporally related outbreak isolates of clone TB. Although phenotypically different, only a few individual SNPs and deletions were detected in the clone TB isolates. Their accessory genome mainly differed in prophage-like DNA elements taken up by one of the strains.

**Conclusions:**

The higher geographical and temporal distance of the clone CHA isolates was associated with an increased intraclonal genome diversity compared to the more closely related clone TB isolates derived from a common source demonstrating the impact of habitat adaptation on the microevolution of *P. aeruginosa*. However, even short-term habitat differentiation can cause major phenotypic diversification driven by single genomic variation events and uptake of phage DNA.

## Background

*Pseudomonas aeruginosa* is a metabolically versatile gamma-proteobacterium that preferentially thrives in aquatic habitats and the rhizosphere [1]. This opportunistic pathogen is the most dominant bacterium causing chronic airway infections in cystic fibrosis (CF) [2] and has become one of the most important causative agents of nosocomial infections, particularly in intensive care units [3].

The 5.2 – 7 Mbp *P. aeruginosa* genome is a mosaic of a conserved core and variable regions of genome plasticity (RGPs) [4]. The core genome is characterized by a conserved synteny of genes [5]. Clonal complexes differ from each other in clone-typical segments of core and accessory genome [6] and a nucleotide divergence in the core genome of 0.5 – 0.7% [7].

Intraclonal whole-genome variation in *P. aeruginosa* has mainly been studied in isolates from CF lungs that had been collected from the same patient longitudinally or at one time point [8-12]. The paired isolates from one patient typically differed due to a few dozens of single nucleotide substitutions (SNPs) and small insertions/deletions (indels) in the core genome, a few RGPs in the accessory genome and occasionally one large deletion or inversion. Close to 1,000 de novo SNPs and indels, however, were gained in hypermutable strains defective in DNA repair [10,12].

Whereas genome microevolution of *P. aeruginosa* in the atypical habitat of the CF lungs has been investigated for several clones, only a single clone has so far been assessed in its genome diversity between strains of unrelated habitat and geographic origin [13]. The two examined clone PA14 strains from California and Germany were found to be of the same genome size and differ from each other in 0.0035% of their nucleotide sequence.

Since these data alone do not allow any general conclusions, we wanted to explore the impact of habitat, history and geographic origin on intraclonal genome diversity of *P. aeruginosa* in more depth. For that purpose two complementary scenarios of habitat differentiation were chosen. The three selected clone CHA strains were isolated from freshwater or CF patients at geographically distant sites within a 15-year period and represent the distant clone strain set. Conversely, the three selected clone TB strains were isolated during a local outbreak and represent the closely related clone strain set. Clones CHA and TB were chosen because we wanted to include the highly pathogenic strains CHA [14] and TBCF10839 [15] in the comparative genome analysis. CHA and TBCF10839 are the only known *P. aeruginosa* strains which can escape killing by leucocytes. TBCF10839 can persist and grow in leucocytes [16], whereas CHA kills leucocytes by type III secretion-dependent oncosis [17-19]. Genome sequencing was expected to provide an explanation why CHA and TBCF10839, but not the other two clone CHA and two TB strains could undermine the major antipseudomonal defence mechanism in humans.

Genome sequencing revealed higher nucleotide divergence and a more variable composition of the accessory genome amongst the less closely related clone CHA strains than amongst the more highly related clone TB strains. Strain-specific SNPs were preferentially detected in habitat-associated fitness loci. Conservation of small non-coding RNA loci followed clone-specific patterns with about 7% (clone TB) or 11% (clone CHA) not conserved. Clone-specific traits were also found for the accessory genomes of the analysed strains, but especially for clone CHA strains which were equipped with several strain-specific DNA elements, the majority of which appeared to be of phage origin. Phage-like DNA also differentiated the accessory genome of the clone TB wound isolate TB63741 from its relatives of CF-origin, indicating that uptake and integration of phage elements is a major driving force of intraclonal diversification of *P. aeruginosa* during adaptation to different habitats.

## Results

### Origin of the *P. aeruginosa* clone CHA and clone TB strains

The clone CHA strains CHA, 491 and PT22 were isolated from sites in Grenoble, Hannover and Mülheim in 1990, 2005 and 1992, respectively. Strain PT22 was isolated from a river, whereas strains CHA and 491 are CF airway isolates. Strain CHA was recovered from a critically ill CF patient with advanced lung disease and chronic *P. aeruginosa* infection [14]. Strain 491 was the first clone CHA isolate from respiratory secretions of a female CF patient with normal lung function [20]. The strain was successfully eradicated from the patient’s airways by antipseudomonal chemotherapy and no further clone CHA strain has since been identified in the patient’s respiratory secretions. The three clone TB strains were isolated from a burn wound (strain TB63741) and two unrelated CF patients (strains TBCF10839 and TBCF121838 [16]) during a local outbreak at Hannover Medical School in summer 1983.

### Shotgun genome sequencing

Fragment libraries of CHA, 491, PT22 and TB63741 were sequenced with the Illumina Genome Analyser II generating 36 bp reads as previously reported for strains TBCF10839 and TBCF121838 [16]. Reads passing quality criteria [10] were mapped to the PAO1 genome sequence ([21]; NCBI sequence NC_002516.2) in order to detect SNPs, indels and PAO1 loci absent in clones CHA and TB. Contigs representing the non-PAO1 loci of the accessory genome were de novo assembled from reads that could not be mapped to the PAO1 reference.

### Comparison of the clone CHA genomes with the PAO1 genome

#### Replacement islands

The *P. aeruginosa* core genome harbours a few loci that are subject to diversifying selection. Clone CHA is equipped with LPS serotype 06, pyoverdine type IIa, a type-a2 flagellin and a novel type I pilin variant.

#### Common SNPs

**Figure 1:**
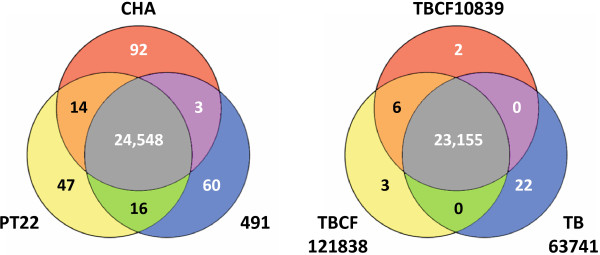
**Venn diagrams of SNPs in clone CHA (left) and clone TB (right).** SNP numbers are based on the alignment to the *P. aeruginosa* PAO1 reference sequence.

**Figure 2:**
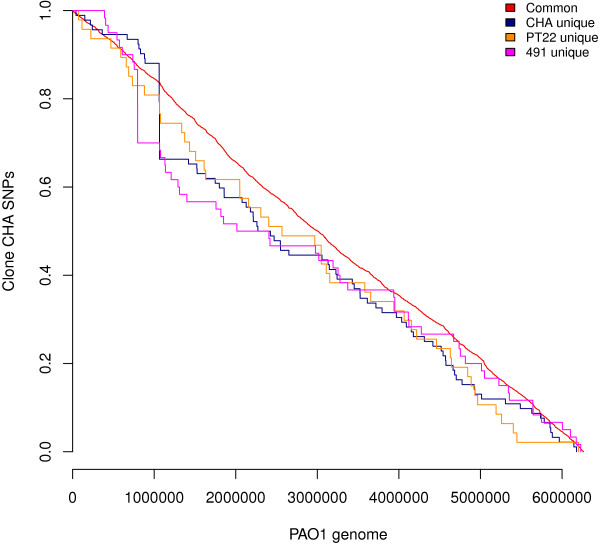
**Kaplan-Meier curves of the proportions of SNPs.** Common synonymous and non-synonymous SNPs found in a) all three clone CHA strains and b) each of the three strains were plotted against genome position in *P. aeruginosa* PAO1. A flat horizontal line indicates that no SNPs were found in that region, while vertical lines illustrate a hotspot of SNPs at this genomic location. The red line shows that SNPs common to all three are evenly distributed throughout the genomes.

The three clone CHA genomes shared 24548 nucleotide exchanges (Figure 1, Additional file 1) compared to the PAO1 reference sequence, which were evenly distributed in the genome (Figure 2). 503 of these lead to a non-conservative replacement of an amino acid as defined by a Dayhoff similarity index [22] of less than 5 (Additional file 2). Table 1 lists these amino acid changes in the 22 proteins whose function have been experimentally demonstrated in *P. aeruginosa* (annotation class I, [23]). Besides a few proteins involved in DNA replication or secondary metabolism, the remaining proteins are transcriptional regulators, members of two-component systems, virulence effectors or are directly or indirectly involved in secretion or biofilm formation. Non-conservative amino acid replacements were neither observed in any enzyme of the core or intermediary metabolism nor in any component of the basic transcriptional or translational apparatus. This comparison of the PAO1 and clone CHA genomes suggests that diversifying selection with impact on protein function has preferentially affected *P. aeruginosa* genes that encode elements for communication with the environment.

**Table 1:** Non-conservative amino acid exchanges (Dayhoff matrix index < 5) in selected proteins* of clone CHA strains

**Locus_tag**	**Name**	**Encoded product**	**aa exchange**
PA0247	PobA	p-hydroxybenzoate hydroxylase	T98M
PA0595	OstA	organic solvent tolerance protein precursor involved in outer membrane biogenesis	M907T
PA0831	OruR	transcriptional regulator of ornithine utilization	W197C
PA1148	ToxA	exotoxin A precursor	F22S
PA1712	ExsB	exoenzyme S synthesis protein B	R52G
PA1717	PscD	type III secretion export protein	V346E
PA1718	PscE	type III secretion export protein	C40G
PA2236	PslF	glycosyl transferase, Psl exopolysaccharide biosynthesis	Y247D
PA3061	PelD	membrane-bound c-di-GMP-specific receptorregulating Pel exopolysaccharide production	Y208H
PA3063	PelB	Pel exopolysaccharide biosynthesis	W791L
PA3344	RecQ	ATP dependent DNA helicase	R571C
PA3805	PilF	pilus biogenesis, outer membrane pilotin for localization and multimerization of secretin PilQ	L243P
PA3810	HscA	molecular chaperone	R285G
PA3910	EddA	extracellular DNA degradation protein	P368L
PA3946	RocS1	sensor of two-component system controlling *cupC* fimbrial and efflux pump gene expression	I399S
PA4085	CupB2	periplasmic chaperone	H242L
PA4086	CupB1	major pilus subunit	Q102T; V154E
PA4776	PmrA	two component regulatory system modulating resistance to cationic antimicrobial peptides	L71R
PA4777	PmrB	two component regulatory system modulating resistance to cationic antimicrobial peptides	Y345H
PA5483	AlgB	two component response regulator controlling alginate biosynthesis	L382R
PA5484	KinB	two component sensor kinase (negative regulation of alginate production, positive regulation of virulence-associated phenotypes)	Y50H
PA5493	PolA	DNA polymerase I	C882R

Exchanges are given in comparison to protein sequences from the PAO1 reference.

*The function of the encoded gene product has been experimentally demonstrated (annotation class I).

#### Indels

Nineteen small indels (< 4 bp) were identified in the coding region of the clone CHA genomes (Table 2), 14 of which were already known from other completely sequenced *P. aeruginosa* strains. The three frameshifts in the last codons of PA3124 and PA4161 or the stop codon of PA5282 are neutral sequence variations and the three in-frame indels in PA2091, PA2302, and PA3462 should modulate the function of the encoded gene products to only minor extent, but the majority of the other 13 out-of-frame indels are probably loss-of-function mutations.

**Table 2:** Small indels in the clone CHA genome compared to the PAO1 genome

**Indel-pos.**^**a**^	**Change**	**Locus_tag**	**Annotation**	**Indel known**
288750	-AT	PA0257	put. integrase/transposase, first ORF of RGP2	no
740420	+C	PA0683	HxcY, type II secretion system protein	yes^1^
995238	+T	PA0912	Hypothetical protein	yes^1^
1060785	+T	PA0977	Hypothetical, phage-like, first ORF in RGP7	no
1116214	+C	PA1029	Hypothetical protein, homology to antitoxin	yes^1^
1697856	+G	PA1559	Hypothetical, part of PmrA regulated operon	yes^2^
1835046	+C	PA1685	MasA, enolase-phosphatase E-1, part of methionine salvage pathway	yes^2^
2301796	-GGC	PA2091	Hypothetical protein	yes^3^
2355772	+G	PA2139	Hypothetical protein	yes^2^
2356683	-C	PA2141	Hypothetical protein	yes^2^
2533912	+GTC	PA2302	AmbE, non-ribosomal peptide synthetase	yes^3^
2753523	+C	PA2452	Similar to enterobactin esterase	yes^2^
3083197	+G	PA2727	Similar DNA helicase	yes^2^
3506327	-C	PA3124	Transcriptional regulator; deletion in last codon	no
3873151	-CCC	PA3462	Sensor kinase of two-component system	yes^1^
4657418	-A	PA4161	FepG, ferric enterobactin transport protein; last codon, no change of coding sequence	yes^4^
4888195	+G	PA4360	Hypothetical, chromosome segregation protein, SMC-like; disruption of start codon	yes^5^
5515497	-A	PA4915	Chemotaxis transducer	no
5945963	+C	PA5282	Major facilitator transporter	no

^a^: position according to PAO1 reference sequence NC_002516.

Indel is listed in the Pseudomonas Genome Database for completely sequenced *P. aeruginosa* genomes: ^1^for strains PA14, 2192, C3719, PACS2, 39016; ^2^for strains PA14, 2192, C3719, PACS2, 39016, PA7; ^3^for strains 2192, C3719, PACS2; ^4^for strains PA14, PACS2, 39016; ^5^for strains PA14, 2192, C3719, PA7, 39016.

Five of the 19 indels are as yet undescribed in the Pseudomonas Genome Database (August 2012). Two of these have no functional consequences as mentioned above (PA3124, PA5282) and one destroys the reading frame of a chemotaxis transducer (PA4915). The remaining two mutations are located in the first ORFs of RGP2 and RGP7, both of which are known to carry clone-specific accessory elements and to be hotspots of genome mobility [13]. The frameshifts inactivate transposase/integrase genes and thus should fix these tRNA-associated genomic islands in the clone CHA genomes.

#### Gain and loss of start and stop codons

The loss of three start and stop codons each and the gain of eight premature stop codons were noted in all three analyzed clone CHA genomes (Table 3). Interestingly another premature stop codon was introduced into ORF PA0977 in all three strains at the same position but by divergent nucleotide exchanges, a transversion in two strains and a transition in the third strain, respectively. Two further nonsense mutations were exclusively identified in strain CHA (Table 3). The mutations affected transcriptional regulators, hypotheticals, glycolate oxidase and Glu-tRNA(Gln) amidotransferase operons. Thus basic bacterial functions of metabolism and translation are impaired or lost in *P. aeruginosa* clone CHA; i.e. glycolate utilization and the transamidation of misacylated Glu-tRNA^Gln^ to correctly charged Gln-tRNA^Gln^.

**Table 3:** SNPs causing gain or loss of start and stop codons in *P. aeruginosa* clone CHA genomes

**Locus_tag**	**SNP-pos.**^**a**^	**SNP**	**Pos. in aa-seq.**	**Length of aa-seq.**	**Annotation**
**Clone CHA [CHA, PT22 and 491] common SNPs – stop codons gained:**					
PA0089	325546	C-T	308	321	Transcriptional activator GpuR
PA1261	1369435	G-A	220	225	Probable transcriptional regulator
PA1427	1553550	G-T	147	188	Hypothetical protein
PA2691	3045894	G-A	87	402	Conserved hypothetical protein
PA4482	5013957	C-A	96	97	Glu-tRNA(Gln) amidotransferase subunit C
PA4982	5598104	G-A	58	999	Probable two-component sensor
PA5342	6010696	C-T	121	267	Probable transcriptional regulator
PA5353	6020049	G-A	356	409	Glycolate oxidase subunit GlcF
**Clone CHA [CHA, PT22 and 491] common SNPs - stop codons lost:**					
PA2456	2756650	A-G	114	114	Hypothetical protein
PA2566	2900372	T-G	396	396	Conserved hypothetical protein
PA6439	5206722	A-G	96	96	Hypothetical protein
**Clone CHA [CHA, PT22 and 491] common SNPs - start codons lost:**					
PA0819	895825	T-C	1	98	Hypothetical protein
PA2778	3136962	A-G	1	292	Hypothetical protein
PA5525	6218101	T-C	1	247	Probable transcriptional regulator
**Divergent nucleotide exchange – stop codon gained:**					
PA0977	1060555	A-C/T	93	108	Hypothetical protein
		A-C in strains PT22 and 491, A-T in strain CHA			
**Strain-specific SNPs in strain CHA only - stop codons gained:**					
PA0734	802084	C-T	52	91	Hypothetical protein
PA5487	6178179	T-A	625	672	Hypothetical protein

^a^: position according to PAO1 reference sequence NC_002516.

#### SNPs shared by two clone CHA strains

Thirty one of 33 SNPs that were found in two, but not in the third CHA strain, are located in two regions of genomic mobility that are prone to horizontal gene transfer [13] suggesting that these SNPs differentiate variants of phage-related sequences. The only two SNPs sensu stricto were identified in intergenic sequences (see Additional file 3).

#### Strain specific SNPs

**Figure 3:**
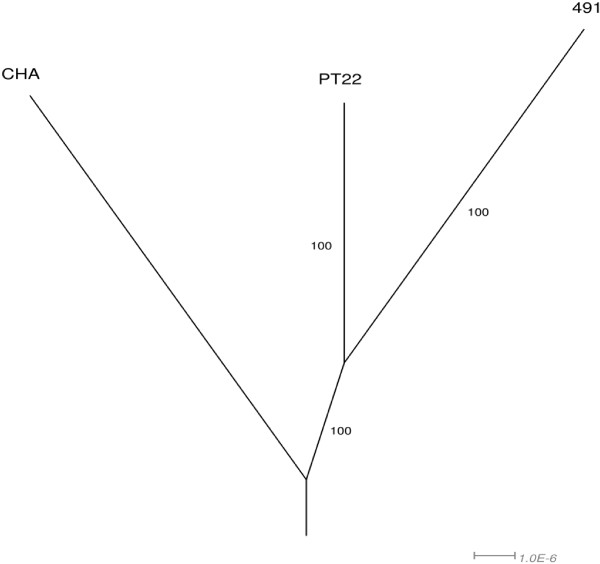
**Phylogenetic network for clone CHA isolates based on identified SNPs.** All SNPs mentioned in Figure 1 were incorporated into three pseudosequences derived from the PAO1 reference sequence by the script SequenceReplacer (available on request from the authors). The network was produced using the uncorrected P distance measure with normalisation followed by the NeighbourNet algorithm in the program Splitstree [62]. The scale indicates the number of substitutions per site. Numbers on the branches are 100 bootstrap resampling values which give a measure of the confidence of the displayed tree topology. A network for clone TB is not shown as the isolates display up to two orders of magnitude less divergence than clone CHA strains, which cannot be visualised appropriately.

The frequency of SNPs shared by two of the three strains was extremely low, but several dozen unique SNPs were found in each of the individual strains indicating some distinct microevolution in the clonally distant strain set (Figure 3). For instance, 47 strain-specific SNPs were identified in the environmental isolate PT22 (Additional file 4). The 34 SNPs in coding regions target genes encoding enzymes, transporters, transcriptional regulators and hypotheticals.

The genome of the CF isolate 491 carries 60 strain specific SNPs (Additional file 4). The clade of strain 491 acquired non-synonymous SNPs in 31 ORFs including genes that should play a role during the colonization of CF airways. Serine-to-asparagine substitutions were present in the two-component response regulator AlgB which activates the transcription of the *algD* alginate biosynthesis operon [24] and the cytoskeleton ATPase MreB which is essential for the maintenance of cell shape, chromosome segregation and polar localization of proteins [25]. The most drastic change was the substitution of arginine by tryptophan R771W in the usher protein CupC3 that is essential for the assembly of CupC1 fimbriae [26]. With 8 of the 60 strain-specific nucleotide exchanges in ORF PA0728, this gene encoding a phage-like integrase was identified as a SNP hotspot in strain 491, and the unique SNPs were not evenly distributed over the whole genome (Figure 2).

Strain CHA carries most unique SNPs among the three sequenced isolates, i.e. 13 intergenic SNPs, 31 synonymous SNPs, 46 non-synonymous SNPs and two SNPs generating a stop codon (Additional file 4). The predicted amino acid sequence was changed in 37 proteins including seven enzymes, six transporters and 15 ones of unknown function. Moreover, the clinically highly virulent strain CHA had acquired missense mutations in seven genes that are key for pathogenicity and adaptation to a habitat such as the CF lungs, i.e. A5G MucA, A651P PelB, R101H ExsA, R156H Tse2, L116F WspA, D514Y PA4036, E721K CbrA. The latter three missense mutations affect the chemotaxis operon WspABCDEF and two sensor kinases of two-component systems. CbrA has been demonstrated to be a global regulator of metabolism, motility, virulence and antibiotic resistance [27-29]. Hence the E721K mutation in CbrA should be a pleiotropic modifier of the bacterial phenotype. Complementation experiments demonstrated that the change of an alanine by glycine in the N-terminus of anti-sigma factor MucA (A5G) leads to the mucoid phenotype, whereas the (by definition) non-conservative exchanges L382R in AlgB and Y50H in KinB of the alginate regulon [30] were not causative for mucoidy in strain CHA (data not shown). The unique ability of strain CHA among functionally characterized *P. aeruginosa* to induce oncosis of neutrophils and macrophages is critically dependent on its active type III secretion system [19]. Whether the undescribed arginine-to-histidine substitution R101H in ExsA, the regulator of the type III secretion regulon, has an effect on the regulon’s activity, is unclear. The non-conservative exchanges in PelB and Tse2 are likely without any consequences for strain CHA. The proteins encoded by the *pel* operon are involved in the biosynthesis of the Pel exopolysaccharide and thus influence biofilm composition and antibiotic tolerance [31], while Tse2, a recently discovered substrate of type VI secretion system of *P. aeruginosa*[32], can inhibit the growth of competing bacterial cells. However, any impact of the mutations in PelB and Tse2 on biofilm stability or competitive fitness, respectively, is uncertain since both are not expressed in the CHA background (data not shown).

Comparable to strain 491, hotspots of strain-specific nucleotide exchanges could also be found in strain CHA, as ORFs PA0982 and PA0977, both located in a region known for genomic instability [33], had acquired nine and three SNPs, respectively.

Twelve, six and five strain-specific SNPs were identified in intergenic regions of strains CHA, PT22 and 491; one of which in each strain affected different sRNAs. Seven strain CHA - specific SNPs were found in the intergenic regions of PA0977-PA0978 (four SNPs) and PA0983-PA0984 (three SNPs) and thus located in the same region prone to genomic instability as 12 of the strains’ unique intragenic SNPs (in PA0977 and PA0982).

#### PAO1-DNA absent in clone CHA strains

The clone CHA genome lacks 117 PAO1 ORFs (2.1% of all ORFs) the majority of which encode pyocins, phage elements or functionally yet uncharacterized gene products (see Additional file 5). Twelve PAO1 ORFs only partially aligned with clone CHA sequence reads indicating that sequence variation is unusually high in these ORFs. All three clone CHA genomes also lack the small non-coding RNA gene (sRNA) *phrD*, that is part of a phage-like insertion in PAO1, and 39 of the 513 intergenic sRNA loci identified recently [34]. Another 21 of these loci were only partially covered by sequence reads of the clone CHA strains (Additional file 6). Intraclonal differences were observed for two sRNA loci. The sRNA pant78 was absent in strain 491 only while pant106 was present in strains PT22 and 491 but absent in strain CHA. Both these pant-sRNAs are located in RGP-insertions in PAO1 (RGP5 or RGP7, respectively) and thus likely contributed to mobile DNA elements.

Strain-specific intragenic deletions of PAO1 coding sequence were observed for two ORFs in strain 491 and one ORF in strain PT22 (Table 4; Additional file 7 Figure S1). Strain CHA showed a 426 bp deletion and, due to that, lacks the last 146 nucleotides of the global regulator *gacS* (PA0928) and the first 278 nucleotides of the adjacent lactate dehydrogenase *ldhA* (PA0927)*.* This two-gene spanning deletion generated a double mutant of key genes of lifestyle and metabolism of *P. aeruginosa*[35,36].

**Table 4:** Strain-specific losses of PAO1 DNA

**Locus_tag**	**Description**
PA0977-0987 (RGP7)	region only partially conserved in all strains; ORFs PA0980-0981 absent in strain CHA only, ORFs PA0986-0987 absent in 491 only
PA0927-0928 (*ldhA, gacS*)	start of *ldhA* (278 nt) and end of *gacS* (146 nt) missing in strain CHA
PA1907	partial deletion (183 nt) in strain 491
PA2136	partial deletion (first 30 nt) in strain 491
PA2177	partial deletion (356 nt) in strain PT22

### The clone CHA accessory genome

#### Accessory DNA elements known from other *P. aeruginosa* clones

**Figure 4:**
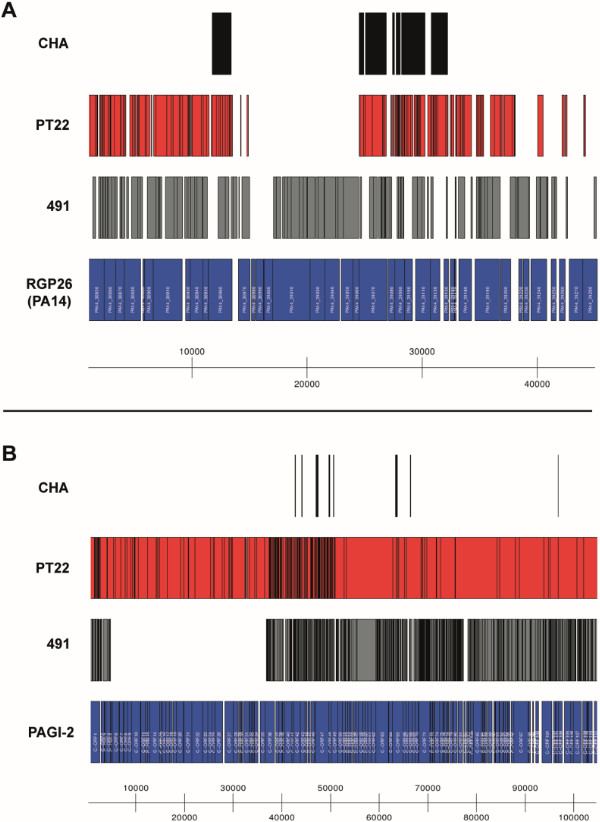
**Diversity of the accessory genome.** As examples, BLAST alignments of de novo assembled not-in-reference accessory genome contigs from all three clone CHA isolates to the PA14 Region of Genome Plasticity (RGP) 26 (panel A) and the PAGI-2 genomic island (panel B) are shown. Contigs from CHA are coloured black, those from PT22 are red and contigs from 491 are grey, while the dark blue boxes represent the annotated ORFs from the *P. aeruginosa* PA14 genome and the PAGI-2 genomic island, respectively. For details on the annotated ORFs, please refer to the respective original publications ([4] for RGP26 from PA14, [39] for PAGI-2). PT22 harbours a complete copy of the PAGI-2 island, while 491 has a partial copy and it is absent in CHA. Figures were produced using the R package Genomegraphs [60].

The clone CHA strains share several genomic islands with the transmissible Liverpool epidemic strain LESB58 [37] (Table 5). CHA, PT22 and 491 harbour copies of LES-prophage 1, LESGI-2 and LESGI-4 of the LES strain and a copy of an RGP29-insertion in the completely sequenced strain PACS2. The three strains moreover share a few ORFs known from insertions in RGPs 6, 9, 27, 36 and 62 in other *P. aeruginosa* genomes [13] (Table 5), although none of these insertions is completely conserved in the clone CHA genomes. Otherwise interstrain diversity is pronounced among the three sequenced clone CHA strains. Each strain carries its specific set of accessory elements. Individual variants were identified for the partially covered RGP26 (Figure 4A) and RGP77 insertions in strain PA14 or PA7, respectively, and for the mobile PAGI-2/pKLC102-type genomic islands. The clone CHA strains also harbour different sets of phage phiCTX-like genes. Variants of this phage either containing or lacking the cytotoxin gene *ctx* have been described for *P. aeruginosa*[38], and apparently such different variants have been acquired by the clone CHA lineage, as the *ctx* gene is conserved in PT22 and 491, but not in strain CHA.

**Table 5:** **Accessory DNA elements from other*P. aeruginosa*genomes detected in strains CHA, PT22, and 491**

**From defined genomic islands**		
**Name**	**No. of ORFs**	**Present parts**
PAGI-2 (RGP29)	111	strain PT22: complete 105 kb island (> 99.9%); strain 491: ORFs C1-4; C36–111 (80 – 100%)
PAGI-5 (RGP7)^a^	121	strain 491: complete 99.4 kb island (> 99.2%)
PAGI-6 (RGP87)	47	**phage CTX-like ORFs 6PG1–28 and 6PG32–38 (86.8 – 100%)**
PAGI-8 (RGP62)	12	**ORFs 8PG1; 8PG7-8 (85.6 – 95.2%)**
pKLC102 (RGP7)^a^	105	strain CHA: ORFs CP1–3; CP12–14; CP27; CP30-31; CP34–44; CP50–54; CP57–83; CP87–89; CP102-103 (94.8 – 99.3%); strain PT22: ORFs CP1–3; CP9–14; CP18–21; CP26–27; CP29–30; CP33–45; CP47–56; CP58–83; CP87–93; CP102-103 (84.3 – 99.5%)
LESGI-2 (RGP85)	18	**complete 31.7 kb island (98.8 – 100%)**
LESGI-3 (RGP27)	115	strain PT22: complete 110.6 kb island (90.4 – 100%); strain 491: PLES_26051–26061; PLES_26211–26221; PLES_26421–27102 (81.7 – 100%)
LESGI-4 (RGP23)^b^	31	**complete 39.4 kb island (97.4 – 100%)**
LES-prophage 1 (RGP3)	19	**complete 14.8 kb island (81.6 – 100%)**
LES-prophage 3 (RGP82)	51	strain 491: homologs to 18 ORFs (88.3 – 98.4%)
LES-prophage 6 (RGP10)	12	strains CHA and 491: PLES_41181 – 41241 (90.7 – 100%); PLES_41191 only partially covered
**From other RGP insertions**		
**RGP (host strains)**	**No. of ORFs**	**Present parts**
RGP6 (2192)	41	**PA2G_05961-05962 (> 99.7%)**
RGP9 (2192)	14	**PA2G_00059-00065; PA2G_00072 (95.1 – 100%)**
RGP26 (PA14)	39	strain CHA: PA14_30960; PA14_31070– 31150 (84.8 – 95.2%); strain PT22: PA14_30850–30960; PA14_31070–31200 (81.1 – 98.6%); strain 491: PA14_30850–30970; PA14_31110–31250 (79.7 – 97.2%)
RGP27 (PACS2)	74	strain 491: PAERPA_01003080–3085; PAERPA_01003110; PAERPA_01003119–3120; PAERPA_01003136–3154 (84.5 – 100%)
RGP29 (PACS2)	10	**complete RGP-insertion (98.5 – 100%)**
RGP35 (2192)	43	strain 491: PA2G_02937–2942; PA2G_02953; PA2G_02956–02957; PA2G_02961–02963; PA2G_02965; PA2G_02969; PA2G_02972-02973 (92.2 – 100%)
RGP36 (PA14)	31	**PA14_15620-15630; PA14_15650-15660 (96.4 – 99.7%)**
RGP42 (2192)	11	strain CHA: PA2G_05286-05290 (97.1 – 99.5%); strain 491: PA2G_05286–05292 (95.4 – 100%)
RGP42 (PA7)	54	strain PT22: PSPA7_5339-5340 (85.1 – 89.1%)
RGP63 (PA7)	72	strain PT22: PSPA7_0075 (86.3 – 90.5%); PSPA7_0108-0114 (> 99.9%)
RGP77 (PA7)	53	strain CHA: PSPA7_3708; PSPA7_3723; PSPA7_3726-3734 (83.4 – 93.3%); strain PT22: PSPA7_3696-3708; PSPA7_3723; PSPA7_3726–3735; PSPA7_3738-3747 (79.5 – 100%); strain 491: PSPA7_3696-3708; PSPA7_3723; PSPA7_3726–3729; PSPA7_3731-3733; PSPA7_3738-3740; PSPA7_3747 (79.3 – 100%)

Present parts printed in bold are conserved in all three clone CHA strains. Pairwise % nucleotide identity of the corresponding sequence contigs is given in brackets.

^a^ majority of assigned contigs mapped on both PAGI-5 and pKLC102 references which share a large set of highly homologous genes.

^b^ contigs also mapped on island PAGI-1, a variant of LESGI-4.

The environmental isolate PT22 is endowed with the largest accessory genome. It carries several ORFs of RGP42 and RGP63 and nearly identical copies of the genomic islands LESGI-3 of strain LESB58 [37] and PAGI-2 of strain C [39] (Figure 4B, Table 5). Strain 491 harbours variants of PAGI-2 and LESGI-3 and phage sequences that are homologous to ORFs in LES-prophages 3 and 6, the latter of which also found in strain CHA.

#### Novel strain-specific genes

ORFs were designated as ‘novel genes’ if they had yet not been described in completely sequenced *P. aeruginosa* genomes deposited in databases by June 1st, 2012. The number of novel genes correlated with the genome size of the strain, i.e. least genes were identified in strain CHA and most genes were detected in strain PT22 (see Additional files 8, 9, 10).

The strain CHA genome incorporated a truncated variant of the *Pseudomonas* phage B3 [40] and an *aacC1* gene that confers resistance to aminoglycoside antibiotics. The *aacC1* sequence contig probably originated from an enterobacterial integron that has the highest homology to the enterobacterial type I integron harboured by plasmid p1658/97 [41].

Annotation uncovered 114 strain-specific ORFs in the CF isolate 491 (see Additional file 10). Most ORFs to which a function could be ascribed encode enzymes of DNA metabolism or mobility or elements of conjugation and type IV secretion. The closest ortholog or homolog was identified for all ORFs in beta- or gamma-proteobacteria that have been classified in the pre-16S rDNA taxonomic era as ‘honorary pseudomonads’ because they share lifestyle, habitat and metabolic versatility with the ‘class I’ pseudomonads *P. aeruginosa*, *P. putida*, *P. fluorescens* and *P. syringae*[42]*.* Twenty-five ORFs are shared with the metal-resistant Burkholderiales *Herminiimonas arsenicoxydans*[43]*.* These genes are part of PAGI-2 like islands harboured by strain 491 (Figure 4B) and the beta-proteobacterium, but none of them is annotated as a metal-resistance contributor.

167 strain-specific ORFs were identified in the aquatic isolate PT22 (see Additional file 9). Like in strain 491, closest orthologs and homologs were detected exclusively among beta- and gamma-proteobacteria, but other genera, namely Acidovorax, Azoarcus, Cupriavidus, Ralstonia (26% of ORFs) and the true pseudomonads (47% of ORFs) were frequent among the closest relatives of PT22 ORFs. The function could be predicted for a larger proportion of ORFs than in the CF isolates, and a greater variety of functions could be addressed which is reflected by a much more diverse spectrum of functional categories/gene ontologies for the PT22-specific ORFs than for those specific for strains CHA or 491 (see Additional file 7 Figure S2). The strain-specific accessory genome of strain PT22 encodes enzymes of lipid and sulphur metabolism, the two-component system *armRS*, a heme lyase and a cytochrome C oxidase and multiple transporters including an efflux pump and a P-type ATPase for heavy metal ions (Additional file 9). Moreover a paralog of the *P. aeruginosa* gene *mvaT* was identified. MvaT belongs to the H-NS family of small DNA-binding proteins that are global regulators of gene expression [44]. Five homologues have been identified in *P. putida* and two homologues *mvaT* and *mvaU* have been identified in the *P. aeruginosa* core genome [45]*. P. aeruginosa* PT22 is thus the first known *P. aeruginosa* strain with three *mvaT* homologues.

### Comparison of the clone TB genomes with the PAO1 genome

In contrast to the analysed clone CHA strains, little intraclonal genomic diversity was observed for the three clone TB strains that were sampled during a local outbreak at Hannover Medical School. As reported earlier, only five individual nucleotide exchanges and one deletion each in a pilus assembly gene could be detected in the two CF airways isolates TBCF10839 and TBCF121838 [16]. Though many phenotypic differences were observed, also the accessory genome differed by only one 81 kb *Ralstonia pickettii* PAGI-2 like genomic island absent in the first but present in the latter isolate [16].

Sequencing of a third clone TB isolate, the wound isolate TB63741, revealed some more intraclonal diversity, but still less than observed for the three clone CHA strains. TB63741 lacked six nucleotide exchanges that were detected for both TB CF isolates, but carried 22 individual SNPs not seen in any of the two CF isolates (Figure 1, Additional file 11). TB63741 did not harbour any deletion in a *pil* gene, but it had acquired a 9-bp in-frame deletion in a two component sensor gene and two frame-shift mutations in a phage gene and in *oprD* (see Additional file 11). The porin OprD transports basic amino acids and peptides but it also takes up the antipseudomonal agent imipenem. Loss-of-function mutations in *oprD* as seen in the clinical isolate TB63741 are a common mechanism of imipenem resistance [46].

Similar to the clone CHA lineage, the conservation of described non-coding sRNA loci does not differ within the clone TB lineage apart from one exception. The sRNA *phrD* and 30 pant-sRNAs are absent in the three genomes, of another 10 pant-sRNA loci significant parts were lacking (see Additional file 6). The phage DNA-associated sRNA pant78, present in both CF-isolates but absent in TB63741 made up the only intraclonal difference regarding sRNAs in clone TB.

Comparison of the sRNA conservation in clonal lineages CHA and TB revealed clone-specific patterns. While *phrD* and 20 pant-sRNA loci from PAO1 were completely absent (and four more partially) in both lineages, clone CHA lacked 17 pant-sRNAs which were present in clone TB. Six pant-sRNAs, however, were absent in clone TB but fully conserved in clone CHA. For another 23 pant-sRNA loci conservation patterns were partially divergent in the two clonal lineages (see Additional file 6). According to that, varying spectra of small non-coding RNA genes in *P. aeruginosa* might contribute significantly to interclonal diversity but only to a small degree to diversity between clonal variants, if sRNA genes are parts of strain-specific acquisition of mobile DNA elements.

Clone TB is endowed with a large accessory genome including the genomic islands PAGI-1, PAGI-2, PAGI-5 and PAGI-6 [16]. The wound isolate TB63741 lacks the 81 kb TBCF121838-specific *R. pickettii* genomic island and numerous phage-like ORFs of phage Pf1 and of genomic island LESGI-1 which were present in both CF isolates. Conversely, TB63741 has incorporated more than 300 kbp that are absent in the two CF strains. Virtually all this DNA is of phage origin including LES-prophage 2 and 3 sequence [37], of which 67.3 or 76.2%, respectively, of the DNA were found in TBCF63741 with nucleotide identities ranging from 80 to 100%. The closest homologues of accessory genome ORFs were found in other *P. aeruginosa* clones, other *Pseudomonas* taxa or in ‘honorary’ pseudomonads (see Additional file 12). The shuffling of phage DNA apparently was the major driving force of microevolution of clone TB during the outbreak.

## Discussion

### Comparison of the sequenced clone CHA and clone TB genomes

This study compared the intraclonal genome diversity of *P. aeruginosa* isolates derived from common and divergent sources*.* Consistent with our expectation higher genomic variation was found among the clonal isolates with a more diverse spatiotemporal origin.

Sequence variation was low among the three clone TB strains that had been sampled in summer 1983 during a local outbreak. The two CF isolates belong to a small epidemic that tripled the prevalence of *P. aeruginosa* – positive patients at the CF clinic [15]. Despite individual profiles of phenotype, strains TBCF10839 and TBCF121838 show only minute differences in their genome sequence [16]. Strain TB63741 was isolated from a patient with severe burns who had been treated at the intensive care unit for burns from which clone TB had initially spread to surgical wards and later to the CF clinic. The ancestors of the TB63741 strain had incorporated numerous phages into the clone TB genome that were absent in the isolates from the CF lungs indicating that highly colonised burn wounds themselves and/or the associated hospital environment had tolerated or favoured the uptake of phages.

The three clone TB isolates had descended from a common source and the individual clades had diverged from each other by at most two years. In contrast, the three sequenced clone CHA isolates were sampled from spatially and temporarily distinct habitats. Correspondingly, the sequence of the core genome and the composition of the accessory genome were significantly more diverse among the three clone CHA than among the three clone TB strains. In particular, the numerous strain specific SNPs in absence of pairwise shared SNPs demonstrate the distinct microevolution of the clone CHA strains (Figure 3). Conversely, shared de novo mutations and comparably very few individual de novo mutations highlight the close relatedness of the two clone TB CF isolates.

The environmental isolate PT22 was endowed with the largest accessory genome of the investigated strains. PT22 was collected from the river Ruhr at a site with substantial anthropogenic pollution and contamination with industrial sewage (Wasserqualität der Ruhr 1992 [47]). Consistent with its source, the genomic islands of PT22 encoded genes for the detoxification of xenobiotics and the efflux of heavy metal ions. PT22 carried a copy of PAGI-2 which also exists in CF isolates and *Cupriavidus metallidurans* CH34 that had been sampled from an industrial site polluted with heavy metal ions [48,49].

The CF airways isolates 491 and CHA were retrieved from patients with the extremes of the general state of health that are feasible with CF as the underlying predisposing condition: The clinically highly pathogenic strain CHA was isolated from a CF patient with end-stage lung disease, whereas strain 491 was recovered from an individual with normal anthropometry and excellent lung function. Strain 491 was eradicated by antipseudomonal chemotherapy and no clone CHA strain has yet been re-isolated from the patient’s respiratory secretions in the last seven years. 491 had gained numerous elements of genomic mobility that may confer some global fitness to the strain, but only a few amino acid substitutions in traits that may facilitate the colonization of CF airways. In other words, the microevolution of the 491 clade does not point to any pronounced selection of the 491 ancestry to accommodate itself to the CF lung habitat.

Conversely, the ancestors of the strain CHA isolate had selected numerous non-conservative amino acid substitutions in elements of chemotaxis, exopolysaccharide biosynthesis, motility and virulence. In addition, the genes *gacS and ldhA* were destroyed by a deletion. The lactate dehydrogenase LdhA has recently been demonstrated in strains PA14 and PAO1 to be indispensible for microcolony formation in biofilms [35]. Hence deletion of the 3’ end of *ldhA* could alter biofilm formation although strain CHA displayed mucoid growth on agar plates (data not shown). The GacS/GacA two-component system controls the reciprocal expression of acute and chronic virulence determinants [34,50]. The deletion of *gacS* should abrogate this control. Consistent with this interpretation, strain CHA strongly expresses the pathways for alginate biosynthesis, a hallmark of a chronic infection, and the virulence effectors and structural elements of type III secretion, a hallmark of an acute infection (mRNA microarray data from bacteria grown to stationary phase, data not shown). Deletions and point mutations in key determinants of virulence and the control thereof thus established a genetic repertoire in the strain CHA isolate that is distinct from 491 and PT22 and should translate into the observed high pathogenic potential in the predisposed human host. This microevolution towards virulence seems to be quite specific for the inhabited CF lungs because strain CHA was inconspicuous in standard *P. aeruginosa* worm and fly infection models [51]. Strain CHA apparently acquired signatures of a host-specific pathogen, whereas the 491 and PT22 clades retained the balance between environmental organism and opportunistic pathogen.

The clone CHA and TB genomes share numerous prophages and genomic islands with the virulent and transmissible LES clone, which has caused substantial morbidity in the CF patient population in the UK [37]. The relatedness of their genomes may explain why these clones are prone to nosocomial spread among predisposed human hosts and why virulent clades with uncommon pathogenicity traits have evolved in these clonal complexes. Subsequent evolvement of pathogenicity arising from such genomic predisposition proceeded differently then in the highly virulent examples TBCF10839 and CHA.

In the case of TBCF10839 only few sequence variations clearly differentiated its genome from that of the other two less virulent TB strains, mainly a loss-of-function mutation in TBCF10839 [52]. While lacking of type IV pili on the surface and being impaired in twitching motility, TBCF10839 was metabolically more active [16], produced more outer membrane transporters and secreted more virulence effectors [53] than its clonal variants. Apparently the loss of PilQ induced a global response in the TB background that is far beyond pilus biogenesis. Any further mutations that are necessary to generate the unique ability of TBCF10839 to grow in neutrophils must have already existed in the clone TB lineage. Strain CHA, however, exhibits numerous strain-specific gain- or loss-of-function mutations in global regulators or key pathogenicity factors that should be involved in the specific virulence features of strain CHA like its capability to cause oncosis of neutrophils [17-19]. Evolvement of the specific pathogenicity traits likely occurred by a series of microevolution events in this case.

## Conclusions

Intraclonal genome diversity in the two investigated strain triplets presented in a low number of strain-specific de novo mutations in the core genome and a variable composition of the accessory genome. Shared SNPs were mainly observed between the two most closely related clone TB isolates from the outbreak. The number of strain-differentiating single nucleotide substitutions ranged from 7 to 154 SNPs for the most and the least related strain pair of clone TB and CHA, respectively. Correspondingly the intraclonal sequence variation of the *P. aeruginosa* core genome was 200- to 3000-fold lower than the interclonal sequence variation of 0.3 – 0.5%. In contrast to the highly conserved core genome a strain-specific signature was noted for the repertoire of phage-related sequences and genomic islands in the distantly related clone CHA strain trio. Strains shared islands and prophages that have first been reported in the transmissible LES strain, but they were distinct in their PAGI-2/pKLC102-type islands that recruit their cargo from the extensive gene pool of the honorary pseudomonads. According to the annotation this cargo as well as the strain specific SNPs confer individual traits on the respective strains to cope with the demands of their habitat from which they were isolated.

## Methods

### Bacterial strains

*P. aeruginosa* strains 491, TBCF10839, TBCF121838 and TB63741 were isolated from patients seen at the Medizinische Hochschule Hannover. Strain PT22 was retrieved from the river Ruhr close to Mülheim. Strain CHA was isolated from a patient seen at the CF clinic in Grenoble. First subcultures were maintained in LB supplemented with 15% (w/v) glycerol at −80°C until use.

### Strain genotyping

*P. aeruginosa* strains were genotyped by a custom-made microarray following the protocol published previously [6].

### DNA preparation

*P. aeruginosa* genomic DNA was prepared from cells grown in LB medium following a protocol optimized for Gram-negative bacteria [54].

### Illumina genome analyser sequencing

After preparing genomic DNA libraries according to the manufacturer’s instructions, sequencing-by-synthesis was performed at GATC-Biotech (Constance, Germany) for each library with an Illumina Genome Analyser II generating 36 bp sequence reads. Illumina Genome Analyser Pipeline Version 0.2 software was applied to qualify reads passing default signal quality filters. Obviously incorrect reads with homooligomers > 13 bases in length (not present in the *P. aeruginosa* genome) or an ‘N’-base call in at least three positions were excluded from the analysis [10]. All sequence data from this study have been submitted to the Sequence Read Archive (SRA) of the EBI (strain TB63741: study accession no. ERP001300; clone CHA strains CHA, PT22 and 491: study accession no. ERP001750).

### Sequence and read alignment

36 bp reads data of the strains were individually mapped to the PAO1 reference genome (NC_002516.2) using the accurate alignment software Novoalign V2.07.00 (Novocraft Technologies, 2010). The command: novoalign –d Indexed_reference_genome –f Reads.fastq –o SAM > out.sam, was used during the mapping to create “sam” formatted alignment files. Two pools of data consisting of the PAO1 mapped and unmapped reads were then extracted directly from the three alignment files using a custom script. Unmapped reads representing non-PAO1 DNA and the mapped reads representing the PAO1 DNA were assigned to not-in-reference and in-reference read pools, respectively.

### Sequence variation sites analysis

Clone CHA strains with genomic positions indicating single nucleotide variants relative to the PAO1 reference were extracted from the novoalign alignment files using SAMtools [55]. The variant call format (vcf) output files generated by SAMtools were further filtered for low quality variants. Variants with minimum coverage of six reads with minimum base calling quality (Q) of 30 at the respective position, a minimum SNP-call quality (QUAL) of 160 (QUAL = −10 log_10_ (probability of wrong call) [56]) and with more than 67% of all quality reads calling the SNP were retained. These variants were then compared against each other to identify sets of strain specific SNPs through the use of an in-house SNP filter pipeline.

The SAMtools derived sequence variants output files were further searched for predictions of small indels. The top candidates (QUAL ≥ 160) were verified by manual inspection of the alignment. Predicted indels were removed that did not pass the following criteria: minimum coverage of more than five high quality reads (Q ≥30 at the candidate position) and more than 95% of reads flag the indel. Predicted indels and SNPs were subsequently annotated using SNPeff version 1.9.5 [57] to identify their effect on coding DNA sequences.

### *De novo* assembly

The not-in-reference pools of sequence reads characterized as Clone CHA accessory genome were assembled to larger contigs with the *de novo* assembler Velvet version 1.0.12 [58]. Commands used during the assembly process are as follows: velveth 63741_cov5_23 23 63741_reads.fas; velvetg 63741_cov5_23 -cov_cutoff 5.0 -max_coverage 300. The assembler parameters were set for a minimum read coverage of 5 and kmer size of 23 to construct reliable contigs. These criteria were set for the analysis as they were demonstrated to maximise the tradeoff between base pairs incorporated and average and maximum contig size after thorough empirical testing. Assembled contigs of strain triplets were aligned against one another by blastn (1e-5 *E*-value threshold) to search for similarity between the sequences. Contigs that lacked similarity with others were designated as strain-specific DNA. These candidates were further validated using alignments of the short read data sets from both other strains using Novoalign. Contigs covered by reads were not considered to be strain-specific.

Validated strain-specific contigs were aligned using blastx against the UniProt database [59] to identify sets of known (present in other *P. aeruginosa*) and novel (not present in other *P. aeruginosa*) genes in their accessory genomes.

### Detection of horizontally transferred genomic elements in clone CHA

Assembled contigs of the three clone CHA strains were aligned against all known *P. aeruginosa* genomic islands and insertions in regions of genome plasticity using blastn (1e-10 *E*-value threshold). Alignment results for all the searches were then visualized by GenomeGraphs [60], an integrated genomic data visualization package for R (http://www.r-project.org) to help determine which of the known horizontally transferred genomic elements are completely/partially present in the three clone CHA strains.

### Check for conservation of predicted sRNAs

Uncovered regions of the reference were extracted from the alignment results for the individual strains and checked for intersection with the 557 sRNA loci described for the PAO1 reference [34]. Complete or partial absence (> 10% not conserved) was confirmed by visual inspection of alignment/coverage for these loci using the Integrative Genomics Viewer [61].

## Competing interests

The authors declare that they have no competing interests.

## Authors’ contributions

JK and BT designed the study. IA, SE and BT provided resources. OKIB and CFD wrote scripts. OKIB, JK and CFD evaluated the sequence data. OKIB, JK, CFD and BT interpreted the sequence data and wrote the manuscript. All authors read and approved the final manuscript.

## Supplementary Material

Additional file 1: Table S1 “SNPs common in clone CHA strains”, contains descriptions of 24560 SNPs detected in all clone CHA strains. Click here for file

Additional file 2: Table S2 “Non-synonymous SNPs in clone CHA strains causing aa-exchanges with Dayhoff-similarity-indices < 5”, contains descriptions of 503 SNPs causing amino acid exchanges. Click here for file

Additional file 3: Table S3 “SNPs shared by two of the three clone CHA strains”, contains lists of SNPs present in two of the three clonal variants. Click here for file

Additional file 4: Table S4 “SNPs specific for strain CHA/PT22/491”, contains three separate lists with SNPs specific for strain CHA, for strain PT22, and for strain 491, respectively. Click here for file

Additional file 5: Table S5 “PAO1 loci absent in clone CHA strains”, describes gene loci from the reference sequence that are absent in the clone CHA strains. Click here for file

Additional file 6: Table S6 “PAO1 ncRNA loci not conserved in clonal lineages CHA and TB”, lists small RNA loci from the reference sequence that are absent in the clone CHA and the clone TB strains. Click here for file

Additional file 7 **Figure S1.**”, a visualization of a strain-specific deletion in the genome of the clone CHA isolate PT22, and “**Figure S2**”, showing gene ontologies / functional categories of strain-specific genes of strains CHA, PT22 and 491. Click here for file

Additional file 8: Table S7 “Alignment of strain CHA-specific accessory genome contigs versus UniProt-database”, contains results of database similarity search for proteins encoded in the strain CHA-specific DNA. Click here for file

Additional file 9: Table S8 “Alignment of strain PT22-specific accessory genome contigs versus UniProt-database”, contains results of database similarity search for proteins encoded in the strain PT22- specific DNA. Click here for file

Additional file 10: Table S9 “Alignment of strain 491-specific accessory genome contigs versus UniProt-database”, contains results of database similarity search for proteins encoded in the strain 491-specific DNA. Click here for file

Additional file 11: Table S10 “TB63741-specific features”, describes SNPs and small indels differentiating strain TB63741 from its clonal variants. Click here for file

Additional file 12: Table S11 “Alignment of TB63741-specific accessory genome contigs versus UniProt-database”, contains results of database similarity search for proteins encoded in the strain TB63741-specific DNA. Click here for file
