# Supplementary material for: Intraclonal genome diversity of Pseudomonas aeruginosa clones CHA and TB
Source: BMC Genomics. 2013 Jun 22;14:416. doi: 10.1186/1471-2164-14-416 (PMC3697988; doi:10.1186/1471-2164-14-416)
Supplement: Additional file 5: Table S5 — “PAO1 loci absent in clone CHA strains”, describes gene loci from the reference sequence that are absent in the clone CHA strains. [file 1471-2164-14-416-S5.doc]

**Additional Table 5: PAO1 loci absent in clone CHA strains**

**PAO1 locus comment**

**from described PAO1-RGP insertions:**

PA0257-0263 complete RGP1 (with ABC transporter component coding genes)

PA0620-0621 part of RGP3 (phage CTX-like genes)

PA0642-0647 complete RGP4 (phage-like DNA)

PA0820-0827 complete RGP6 (phage-like DNA)

PA1239-1241 complete RGP48 (metabolic genes and transcriptional regulator)

PA1368-1372 complete RGP13 (phage-like DNA)

PA1935-1936, PA1939 parts of RGP52 (with endonuclease coding gene)

PA2036-2037 part of RGP20 (with metabolic gene)

PA2100-2106 complete RGP21 (metabolic genes, transporter and regulator)

PA2218-2228 part of RGP23 (beta-lactamase-like gene and regulators)

PA2461 part of RGP25 (similar to secreted effector coding gene)

PA2730-2736 part of RGP28 (type I restriction modification system genes

PA2818-2819 complete RGP29 (with aminoglycoside response regulator gene)

PA3367 complete RGP58 (gene of unknown function)

PA3497-3514 complete RGP34 (metabolic and ABC transporter genes)

PA3866-3869 complete RGP37 (pyocin and phage-like genes)

**other loci:**

**completely absent:**

PA0053 similar to gene for prophage maintenance system killer protein

PA0457.1 membrane protein coding gene

PA0497-0499 with pilus assembly chaperone gene

PA0574 similar to fic family protein coding genes

PA0631-0641 phage-like genes

PA0648 phage-like gene

PA1471-1472 with acetyltranferase-like gene

PA2073 transporter component coding gene

PA3487-3488 with phospholipase D coding gene

PA5264-5265 transmembrane protein coding genes

**only partially conserved:**

PA0145 similar to nucleoside 2-deoxyribosyltransferase coding gene

PA0573 similar to nuclease coding genes

PA1149 and PA1150 with pys2 pyocin coding gene

PA2074 amidohydroylase gene, likely polyamine biosynthesis gene

PA3290 and PA3291 genes of unknown function

PA3486 similar to type VI secretion system protein coding gene

PA4075 probable methyltransferase coding gene

PA4514 gene coding probable siderophore receptor protein

PA5089 lipid metabolism gene

PA5266 similar to type VI secretion system protein coding gene
