# Supplementary material for: Intraclonal genome diversity of Pseudomonas aeruginosa clones CHA and TB
Source: BMC Genomics. 2013 Jun 22;14:416. doi: 10.1186/1471-2164-14-416 (PMC3697988; doi:10.1186/1471-2164-14-416)
Supplement: Additional file 7 — Figure S1.”, a visualization of a strain-specific deletion in the genome of the clone CHA isolate PT22, and “Figure S2”, showing gene ontologies / functional categories of strain-specific genes of strains CHA, PT22 and 491. [file 1471-2164-14-416-S7.pdf]

Supplemental Figures and Legends

A strain-specific deletion in the genome of isolate PT22 was visualized by displaying read alignment results in the Integrative Genome Viewer: Thorvaldsdottir H, Robinson JT, Mesirov JP: **Integrative Genomics Viewer (IGV): high-performance genomics data visualization and exploration.** *Brief Bioinform* 2012 Apr 19 doi: 10.1093/bib/bbs017.

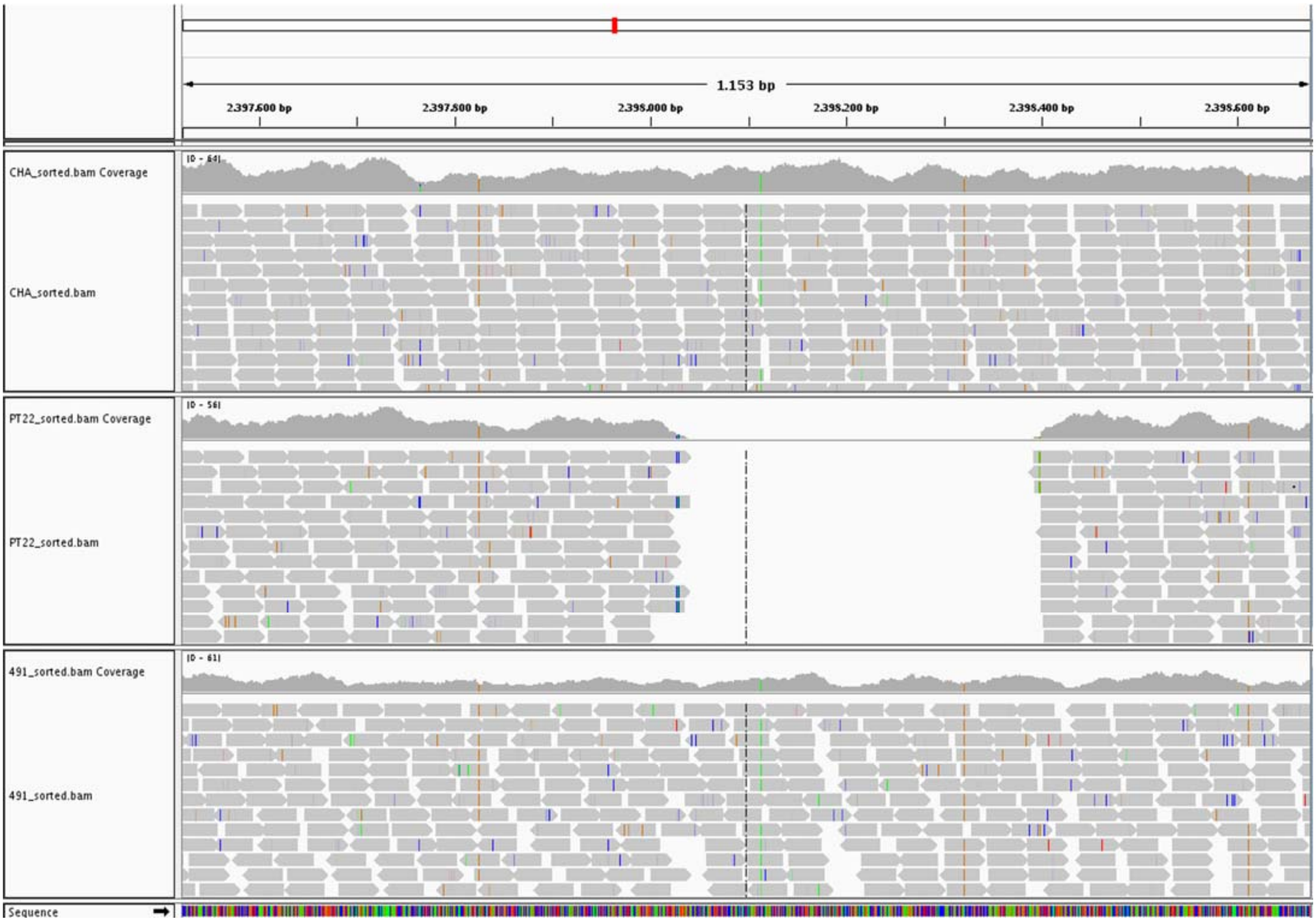

Supplementary Figure S1: Visualization of a strain-specific deletion in PT22. Alignments of strain CHA (top), PT22 (middle) and 491 (bottom) reads to the PAO1 reference (NC_002516.2) are displayed by the Integrative Genomics Viewer (Thorvaldsdottir et al., 2012). Positions 2,397,600 – 2.398,600 of the reference are shown, which are fully covered in CHA and 491 while in PT22 a block of 356 bp is uncovered.

The GO gene ontology based figures for Clone CHA were created using Treemaps: Tennekes, M., de Jonge, E. (2011). Top-down data analysis with treemaps. Proceedings of the International Conference on Information Visualization Theory and Applications, IVAPP 2011, Algarve, Portugal.

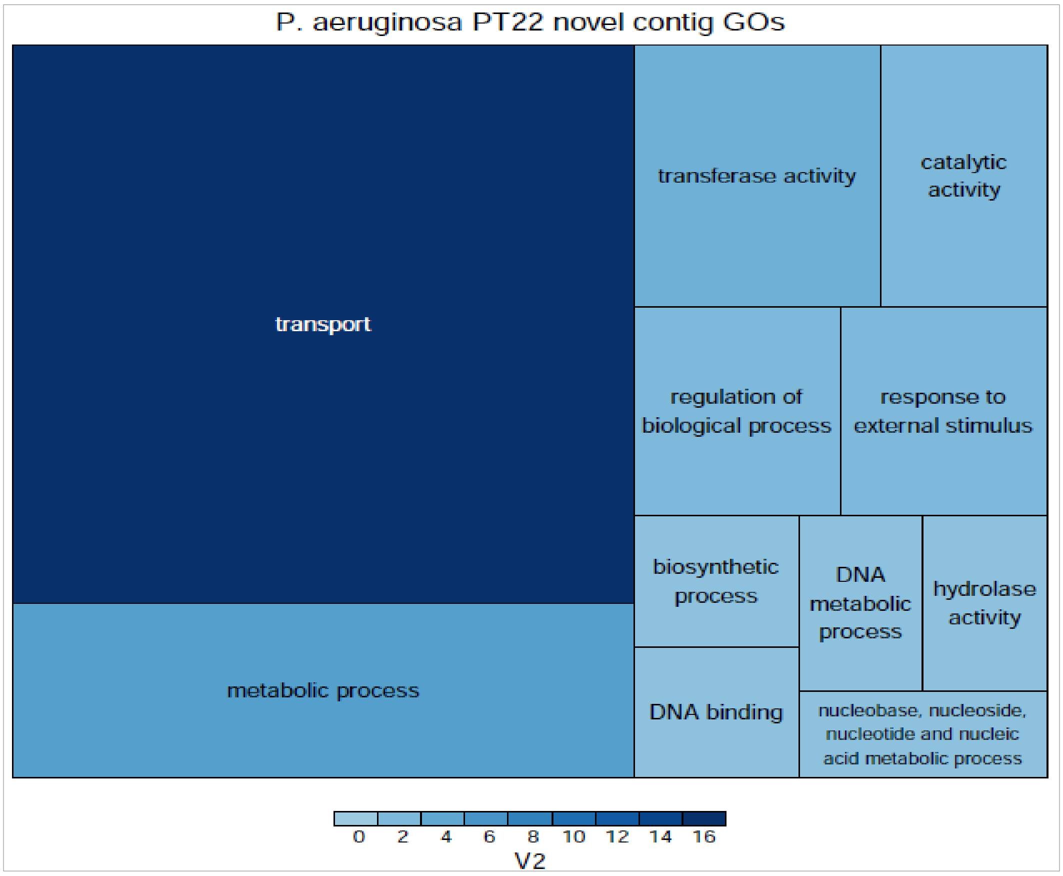

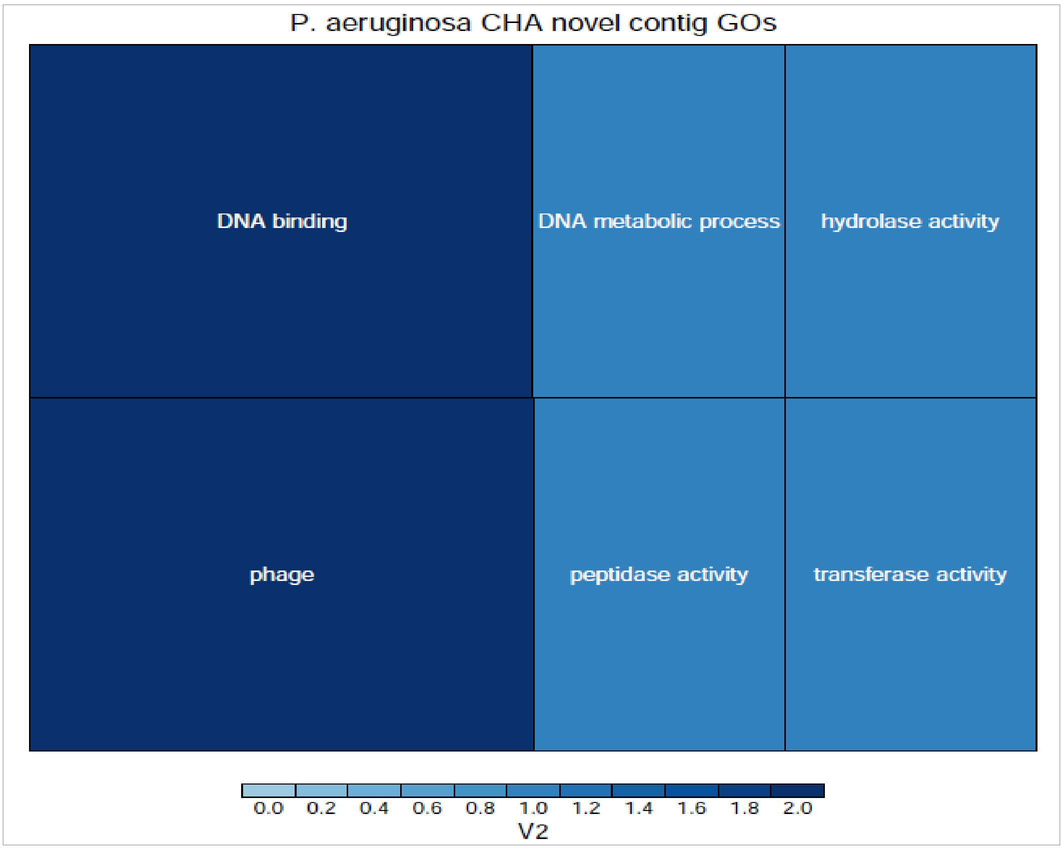

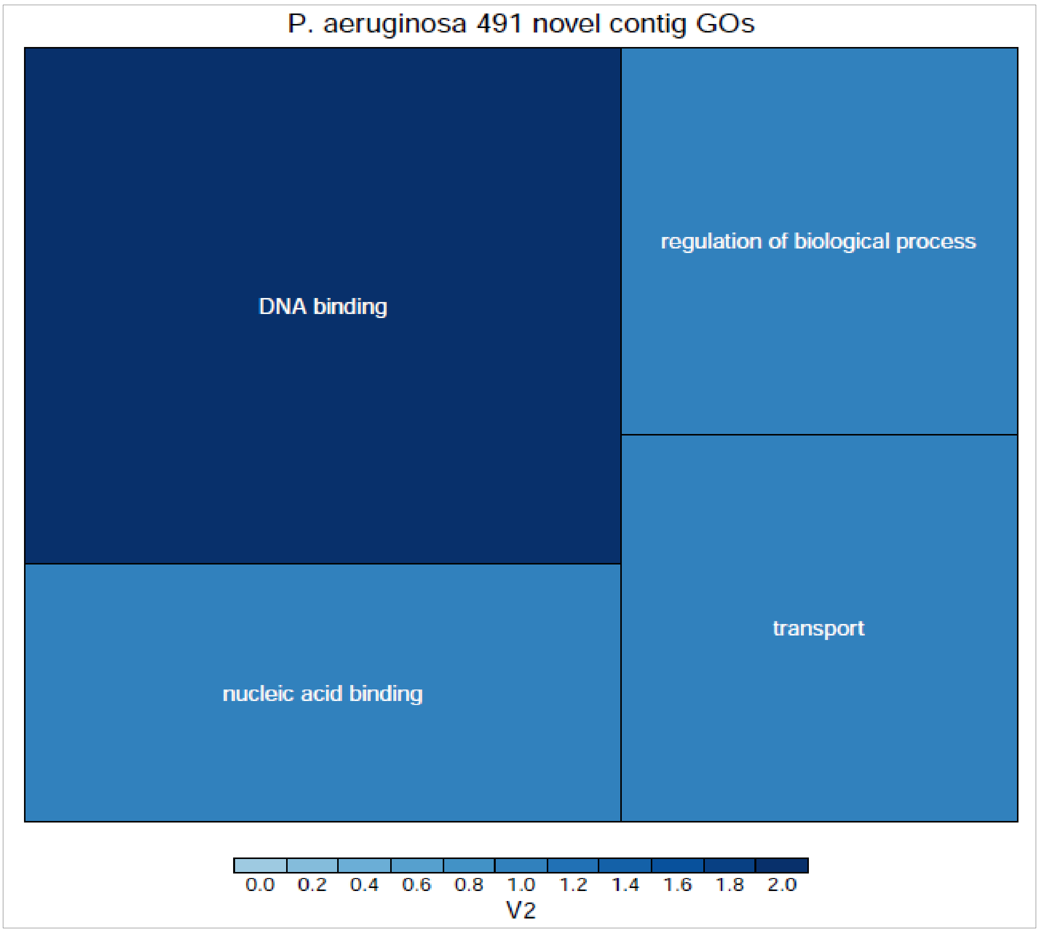

Supplementary Figure S2: Treemaps (Tennekes et al. 2011) of functional categories as defined by the GO gene ontology for all strain-specific contigs from the three accessory genomes. A larger and darker square indicates a higher proportion genes of that functional category. PT22 has the most diverse unique accessory genome, while those of CHA and 491 are very restricted. Only those contigs containing genes with GO terms were included.
